# Association between triglyceride glucose index and adverse cardiovascular prognosis in patients with atrial fibrillation without diabetes: a retrospective cohort study

**DOI:** 10.1186/s12944-025-02447-3

**Published:** 2025-01-25

**Authors:** Aobo Gong, Ying Cao, Zexi Li, Wentao Li, Fanghui Li, Yao Tong, Xianjin Hu, Rui Zeng

**Affiliations:** Department of Cardiology, West China Hospital, Sichuan University West China School of Medicine, 37 Guoxue Road, Chengdu, Sichuan 610041 China

**Keywords:** Atrial fibrillation, Triglyceride glucose index, Major adverse cardiovascular events, Risk prediction, Clinical prognosis

## Abstract

**Background:**

Atrial fibrillation (AF) is the most prevalent arrhythmia encountered in clinical practice. Triglyceride glucose index (Tyg), a convenient evaluation variable for insulin resistance, has shown associations with adverse cardiovascular outcomes. However, studies on the Tyg index’s predictive value for adverse prognosis in patients with AF without diabetes are lacking.

**Methods:**

This retrospective study utilized electronic medical records to collect data on patients with AF hospitalized at West China Hospital from January to June 2020. Participants were categorized into three groups based on their Tyg index levels. The primary outcome, major adverse cardiovascular events, included cardiac death, stroke, and myocardial infarction. Kaplan–Meier curve, Cox proportional hazards regression model, and restricted cubic spline were employed to explore the relationship between the Tyg index and outcomes. The predictive performance of the CHA2DS2-VASc model was evaluated after incorporating the Tyg index.

**Results:**

The study comprised 864 participants (mean age 67.69 years, 55.32% male, 57.52% paroxysmal AF). Patients with high Tyg index had a significantly higher risk of developing major adverse cardiovascular events (MACE) (*P* < 0.001, hazard ratio: 2.05, 95% confidence interval:1.65–2.56). The MACE risk in the middle Tyg group was similar to that in the low Tyg group (*P* = 0.1) during the 48-month follow-up period. However, focusing on the last 24 months revealed a higher MACE risk (*P* = 0.015) in the middle Tyg group. The restricted cubic spline analysis revealed an S-shaped correlation between Tyg and MACE. The CHA2DS2-VASc model combined with the Tyg index showed improved predictive performance and net benefit.

**Conclusions:**

A high Tyg index is associated with poorer prognosis in patients with AF without diabetes. Integrating the Tyg index into the CHA2DS2-VASc model may enhance its predictive performance, offering clinical utility.

**Supplementary Information:**

The online version contains supplementary material available at 10.1186/s12944-025-02447-3.

## Introduction

Atrial fibrillation (AF) is the most prevalent arrhythmia and is associated with poor cardiovascular and cerebrovascular prognosis [1]. Patients with AF and diabetes are susceptible to adverse cardiovascular events [2]. Insulin resistance is a pivotal factor in the occurrence and progression of diabetes. This condition frequently manifests early on and persists, giving rise to associated metabolic abnormalities and increasing cardiovascular risk among patients with AF [3]. Moreover, insulin resistance is believed to promote oxidative stress and emerges as a contributory factor to the development of left atrial fibrosis [2, 4].

The gold standard for diagnosing insulin resistance is the euglycemic-hyperinsulinemia clamp test, which is not only complex but also costly [5]. The Homeostatic Model Assessment of Insulin Resistance (HOMA-IR) is widely used; however, cardiovascular doctors rarely take the initiative to conduct such tests. The triglyceride glucose index (Tyg), calculated using blood glucose and blood triglycerides, is a more convenient alternative indicator for insulin resistance in clinical practice, achieving sensitivity and specificity comparable to HOMA-IR [6]. Considerable evidence indicates the correlation between the Tyg index and cardiovascular disease. In patients with hypertension, a high Tyg index indicates an increased risk of chronic kidney disease and myocardial infarction [7, 8], and it also has the capability to predict the risk of stroke [9]. Moreover, recent evidence has suggested that the occurrence of AF, as well as its recurrence after catheter ablation, can be predicted by the Tyg index [10–12]. In addition, certain drugs that control blood glucose and lipids have shown promise in reducing AF occurrence [13, 14] and improving the prognosis of AF patients [15, 16]. However, studies on the association between the Tyg index and poor cardiovascular prognosis in patients with AF without diabetes are lacking.

This study aimed to assess whether the Tyg index can serve as a biomarker for predicting adverse cardiovascular and cerebrovascular outcomes in AF patients without diabetes. By incorporating the Tyg index into the well-established CHA2DS2-VASc score, which is a clinical prediction model for patients with non-valvular AF, this study aids the development of a novel cardiovascular event prediction system and assesses its potential for clinical application.

## Methods

### Study population

This was a retrospective, observational, single-center cohort study. This study was conducted using data from patients who were hospitalized at West China Hospital of Sichuan University from January to June 2020. Patients who met the following criteria were included: (1) aged ≥ 18 years and (2) diagnosed with AF during admission. Exclusion criteria included: (1) patients who died during hospitalization; (2) patients who were excluded from a definitive diagnosis of AF during hospitalization based on the European Society of Cardiology diagnostic criteria for AF [17]; (3) patients without at least one follow-up visit; (4) patients previously or currently diagnosed with diabetes; (5) patients with severe valvular diseases or a history of valve surgery or patients who have undergone congenital heart disease surgery; (6) patients with concurrent malignant tumors; and (7) patients who had not undergone blood glucose or triglyceride testing during hospitalization.

**Fig. 1:**
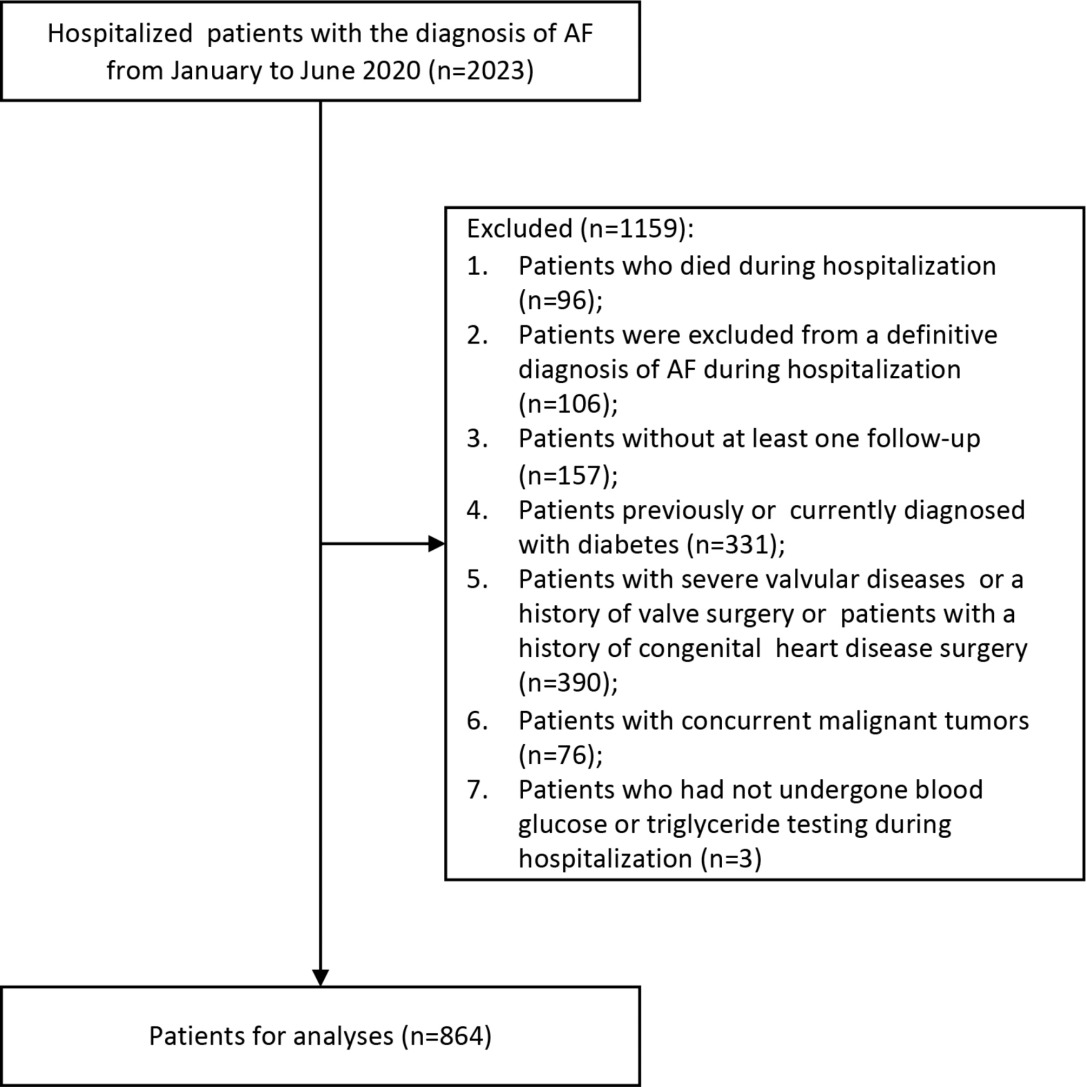
Study flow chart. AF = atrial fibrillation

Ultimately, 864 cases were analyzed, of which 308 participants underwent AF catheter ablation during hospitalization. Overall, 1159 cases were excluded for meeting the exclusion criteria, among which patients with severe valvular disease or cardiac surgery were excluded due to their serious impact on the cardiovascular prognosis. Additionally, patients with malignant tumors that may cause serious metabolic disorders were excluded. The detailed research flowchart is presented in Fig. 1.

### Basic data collection

Clinical data such as demographic characteristics, comorbidities, admission blood pressure, blood glucose, blood triglycerides, N-terminal pro B-type natriuretic peptide (NT-pro BNP), glutamyl transpeptidase, and other laboratory tests were collected from electronic medical records. Additionally, echocardiography data, such as left ventricular maximum diameter (LVD) and left ventricular ejection fraction (LVEF). AF types were classified as paroxysmal or persistent, depending on whether the AF rhythm could be sustained for 7 days. The Tyg index was obtained using the formula: Ln [blood triglyceride (mg/dL) × blood glucose (mg/dL)/2]. The CHA2DS2-VASc score was computed based on the presence of heart failure (HF), hypertension, diabetes, coronary artery disease (CAD)/peripheral artery disease (PAD), and female sex, each contributing one point. One point was allocated for 65–74 years old, and two points for ≥ 75 years old. Stroke/transient ischemic attack (TIA)/systemic embolism contributed two points. Medication information for all patients was extracted from discharge orders. The data were extracted by two cardiology clinicians, and any disputes were resolved through group discussions.

### Study outcome and follow-up

The primary outcome of the study is a composite of major adverse cardiovascular events (MACE), including cardiac death, stroke, and myocardial infarction. The follow-up period commenced upon discharge of the participants and concluded in March 2024. Three trained cardiology clinicians followed up with all participants through electronic medical records, outpatient services, online services, and telephone calls.

### Statistical analysis

Based on their Tyg index level, all participants were assigned to three equal groups with cutoff values set at 8.493, 9.023 (First group: Tyg ≤ 8.493; Middle group: Tyg 8.494–9.022; Third group: Tyg: ≥9.023). Descriptive statistics were used to describe the basic data, presenting continuous variables as mean (standard deviation) and categorical variables as frequency (percentage). The differences between categorical and continuous data were examined using chi-square tests and analysis of variance, respectively.

To evaluate the connection between MACE and the Tyg index, Kaplan–Meier curves were used for visualization, and the log-rank test was conducted to evaluate the differences. Landmark analysis was also performed to assess outcomes between 24 and 48 months. Schoenfeld residual test was used to verify the assumption of proportional hazards. Except for age and high-density lipoprotein, all variables satisfied the assumption of proportional hazards. Therefore, age was categorized into two groups (under and over 65 years) and high-density lipoprotein into two groups (under and over 1 mmol/L) for subsequent Cox regression and model building. A restricted cubic spline was conducted, adjusting for participants’ sex and age. The Akaike information criterion (AIC) of models with 3, 4, 5, and 6 knots were calculated, and the models with lowest AIC were selected. Hazard ratios (HR) and 95% confidence intervals (CI) of the exposures for MACE were calculated using univariate Cox proportional hazards models, considering for variables including catheter ablation, laboratory indicators, Tyg index, and echocardiography data. Regarding variables with missing data, the samples with missing values were excluded. Subsequently, a multivariate Cox regression model adjusting for CHA2DS2-VASc variables (sex, age, hypertension, HF, stroke/TIA/systematic embolism, and CAD/PAD) and catheter ablation was conducted. In addition, subgroup analysis was conducted based on gender, AF type, age (< 65 years old or ≥ 65 years old), catheter ablation, and HF.

The Tyg was added as categorical data into the CHA2DS2-VASc variables to form a new model (model 1). The AIC was chosen to estimate the fitting performance of the models, and models with a smaller AIC were considered to have a relatively better performance than models with a higher AIC. In addition, the differences between model 1 and the traditional CHA2DS2-VASc model (model 2) were visualized using a time-dependent area under curve (AUC), with AUC comparisons made at 12-month intervals (12, 24, 36, 48 months). Without considering the impact of time, the Receiver Operating Characteristic (ROC) curves were obtained and compared for both models. Additionally, scores of 0, 1, and 2 were assigned to each Tyg group, integrating these scores into the CHA2DS2-VASC score. Subsequently, a cutoff value of 4 was selected for this scoring system according to the principle of minimum *P*-value to evaluate the predictive efficacy. Moreover, a clinical decision curve analysis (DCA) was generated to assess the clinical applicability of model 1 compared to model 2 [18].

Statistical analyses were performed using R software (version 4.1.2, https://www.r-project.org/), with statistical significance set at *P* ≤ 0.05.

## Results

### Study population

In the final analysis, 864 participants were accounted for, having an average age of 67.69 years. Of these participants, 55.32% were male, and 57.52% had paroxysmal AF. Over a median follow-up time of 47 months (95% CI: 46.8–47.19),148 participants (17.13%) developed MACE.

Among the participants, 308 underwent catheter ablation treatment for AF during the admission period, with most falling into the first group (≤ 8.493) and middle group (8.494–9.022). Participants in the middle group (8.494–9.022) and third group (≥ 9.023) had a higher body weight compared to those in the first group (≤ 8.493). Additionally, the participants in third group (≥ 9.023) had a higher incidence of HF, larger LVD, lower LVEF, and higher NT-pro BNP. The participants with high Tyg index showed elevated levels of low-density lipoprotein cholesterol, glutamyl transpeptidase, serum uric acid, serum creatinine, and total cholesterol and along with lower levels of high-density lipoprotein cholesterol. Moreover, patients in the third group (≥ 9.023) had lower rates of oral anticoagulant (OAC) use. Table 1 presented the additional baseline data.

**Table 1:** Baseline participants characteristics

	First group Tyg ≤ 8.493	Middle group Tyg 8.494–9.022	Third group Tyg ≥ 9.023	*P*.value
	*N* = 288	*N* = 288	*N* = 288	
Mean age (SD), y	67.4 (14.2)	67.4 (14.3)	68.2 (13.2)	0.734
Male sex (%)	159 (55.2)	161 (55.9)	158 (54.9)	0.968
Paroxysmal AF (%)	158 (54.9)	166 (57.6)	173 (60.1)	0.449
Catheter ablation (%)	120 (41.7)	119 (41.3)	69 (24.0)	< 0.001
Weight (SD), kg	61.1 (12.5)	63.7 (13.5)	64.7 (13.0)	0.006
Unknown (%)	20 (6.9)	27 (9.4)	32 (11.1)	
Height (SD), m	1.62 (0.09)	1.63 (0.09)	1.62 (0.09)	0.584
Unknown (%)	10 (3.5%)	19 (6.6)	17 (5.9)	
Systolic pressure (SD), mmHg	130 (19.5)	129 (19.9)	129 (23.0)	0.733
Diastolic pressure (SD), mmHg	80.7 (13.7)	80.5 (13.5)	80.0 (14.7)	0.798
CHA2DS2-VASc (SD)	2.84(1.86)	2.82(1.92)	3.18(1.94)	0.038
Comorbidities (%)				
Hypertension	150 (52.1)	133 (46.2)	145 (50.3)	0.346
Heart failure	86 (29.9)	95 (33.0)	118 (41.0)	0.015
Stroke/TIA	47 (16.3)	49 (17.0)	54 (18.8)	0.730
Systemic embolism	1 (0.4)	6 (2.1)	12 (4.2)	0.007
Coronary artery disease	60 (20.8)	59 (20.5)	85 (29.5)	0.015
Peripheral artery disease	25 (8.7)	13 (4.5)	17 (5.9)	0.114
COPD	33 (11.5)	23 (8.0)	30 (10.4)	0.361
Sleep disorders	11 (3.8)	14 (4.9)	9 (3.1)	0.559
Hyperthyroidism	4 (1.4)	10 (3.5)	1 (0.4)	0.014
Hypothyroidism	12 (4.2)	13 (4.5)	9 (3.1)	0.672
Laboratory tests:				
GGT(SD), IU/L	48.8 (68.7)	52.3 (80.9)	77.2 (91.1)	< 0.001
Cholesterol (SD), mmol/L	3.76 (0.90)	3.99 (0.92)	4.29 (1.07)	< 0.001
LDL(SD), mmol/L	2.09 (0.74)	2.29 (0.78)	2.51 (0.92)	< 0.001
HDL(SD), mmol/L	1.31 (0.38)	1.18 (0.31)	1.04 (0.34)	< 0.001
SUA(SD), umol/L	336 (113)	365 (123)	404 (144)	< 0.001
SCR(SD), umol/L	93.4 (63.3)	98.3 (93.4)	132 (143)	< 0.001
NT-pro BNP(SD), ng/L	2313 (4073)	2588 (5052)	5841 (9057)	< 0.001
Unknown (%)	40(13.9)	29(10.1)	25(8.7%)	
Echocardiogram data:				
LAD(SD), mm	41.7 (8.72)	40.8 (7.84)	41.0 (7.64)	0.446
Unknown (%)	11(3.8)	19(6.6)	32(11.11)	
LVD(SD), mm	48.3 (6.05)	47.7 (6.29)	49.5 (8.48)	0.010
Unknown (%)	12(4.2)	19(6.6)	32(11.11)	
RAD(SD), mm	41.9 (8.71)	39.6 (8.18)	40.3 (7.78)	0.005
Unknown (%)	16(5.6)	28(9.7)	51(17.7)	
RVD(SD), mm	22.4 (3.07)	22.0 (3.08)	22.0 (3.46)	0.302
Unknown (%)	12(4.2)	22(7.6)	45(15.6)	
LVEF(SD), %	61.2 (10.4)	61.4 (11.3)	57.9 (14.1)	0.001
Unknown (%)	11(3.8)	20(6.9)	33(11.5)	
Medications, %				
OAC only	188 (65.3)	187 (64.9)	148 (51.4)	0.001
Antiplatelet only	23 (8.0)	24 (8.3)	28 (9.7)	0.736
OAC + Antiplatelet	20 (6.9)	21 (7.3)	24 (8.3)	0.806
ARNI/ACEI/ARB:	64 (22.2)	62 (21.5)	62 (21.5)	0.973
β-blocker:	80 (27.8)	93 (32.3)	95 (33.0)	0.341
Statin:	69 (24.0)	87 (30.2)	88 (30.6)	0.141

Tyg: triglyceride glucose index; SD: standard deviation; AF: atrial fibrillation; CHA2DS2-VASc: heart failure, hypertension, diabetes, coronary artery disease/peripheral artery disease and female with one point each, one point for 65–74 years old, and two points for ≥ 75 years old, stroke/transient ischemic attack/systemic embolism with 2 points; TIA: transient ischemic attack; COPD: chronic obstructive pulmonary disease; GGT: glutamyl transpeptidase; LDL: low density lipoprotein; HDL: high density lipoprotein; SUA: serum uric acid; SCR: serum creatinine; NT-pro BNP: N-terminal pro B-type natriuretic peptide; LAD: left atrial anterior-posterior diameter; LVD: left ventricular maximum diameter; RAD: right atrial maximum diameter; RVD: right ventricular maximum diameter; LVEF: left ventricular ejection fraction; OAC: oral anticoagulation; ARNI: Angiotensin Receptor & Neprilysin Inhibitor; ACEI: angiotensin converting enzyme inhibitor; ARB: angiotensin receptor blocker

### Association of Tyg Index with MACE

**Fig. 2:**
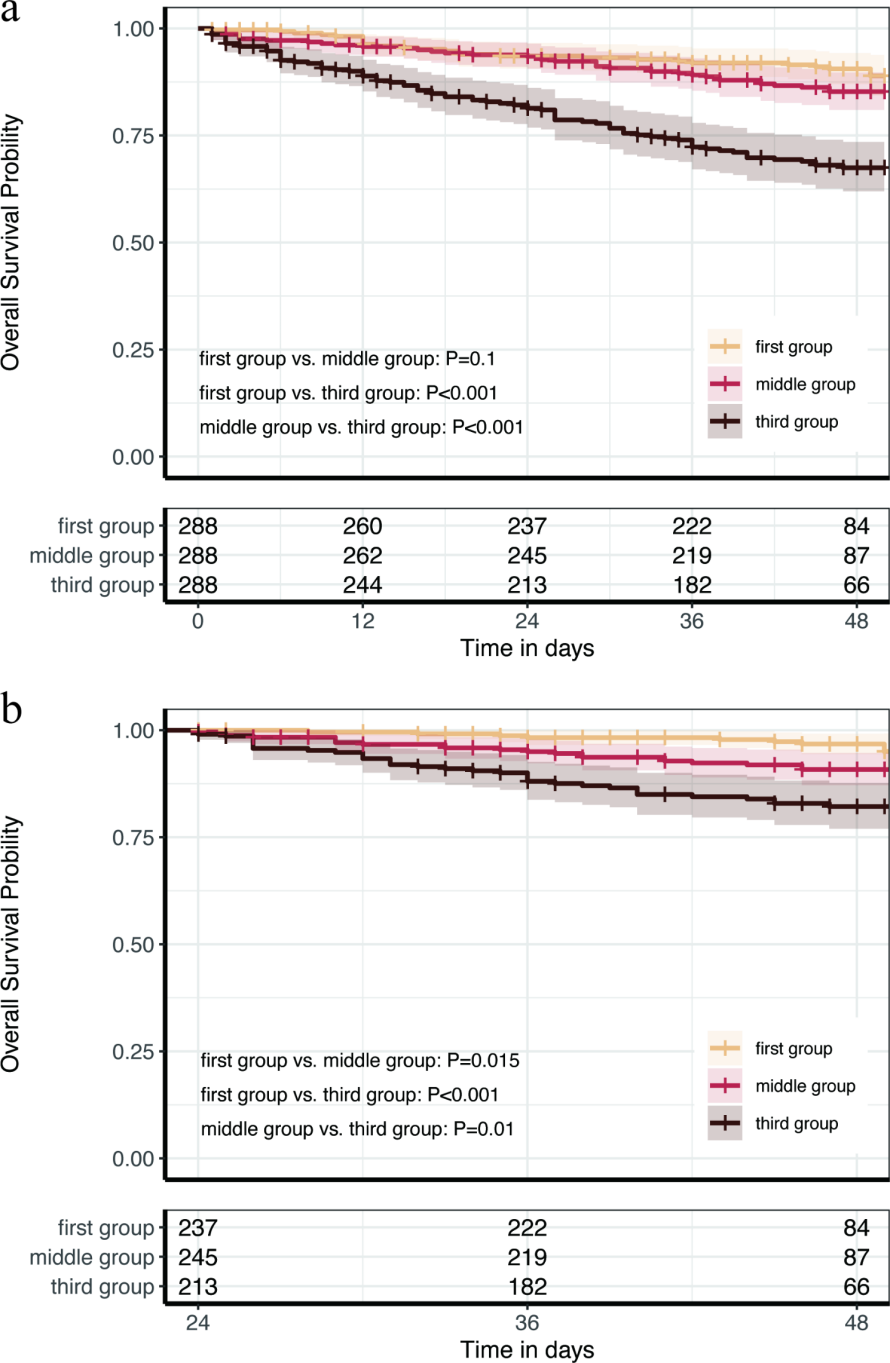
a: Kaplan–Meier curve of major adverse cardiovascular events. b: Kaplan–Meier curve of major adverse cardiovascular events with a 24-month landmark. First group: triglyceride glucose index ≤ 8.493; Middle group: triglyceride glucose index 8.494–9.022; Third group: triglyceride glucose index ≥ 9.023

The third group (≥ 9.023) had a significantly higher occurrence rate of MACE compared to the first (≤ 8.493) and middle group (8.494–9.022) (*P* < 0.001). However, the survival rate of MACE over 4 years of follow-up in the middle group (8.494–9.022) was similar to that in the first group (≤ 8.493) (*P* = 0.1) (Fig. 2a). The four-year overall survival rates were 67.46% (95% CI, 61.95–73.47%) in the third group (≥ 9.023), 85.24% (95% CI, 80.98–89.72%) in the middle group (8.494–9.022), and 90.54% (95% CI, 86.99–94.23%) in the first group (≤ 8.493). After setting a 24-month landmark, the first group (≤ 8.493) exhibited a higher survival rate compared to the middle Tyg group (8.494–9.022) (*P* = 0.015) (Fig. 2b).

**Fig. 3:**
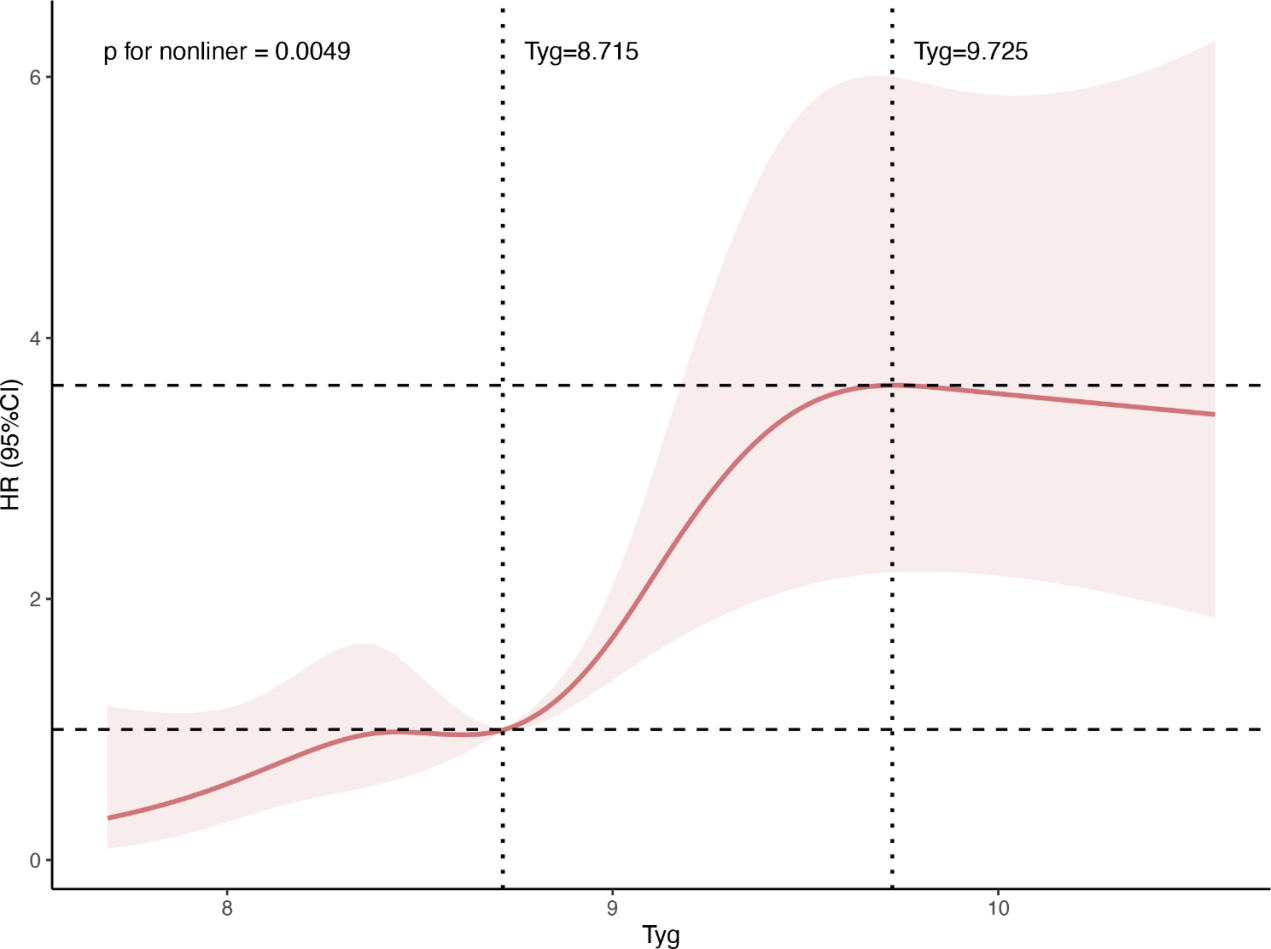
Association of Tyg index with major adverse cardiovascular events evaluated by restricted cubic spline. Tyg index = triglyceride glucose index; HR = hazard radio

To construct a restricted cubic spline model, the AIC for models with 3, 4, 5, and 6 knots was calculated. The AIC values for models with 3, 4, 5, and 6 knots were 1837.197, 1834.069, 1832.532, and 1834.617, respectively. Consequently, the model with 5 knots (5th, 35th, 50th, 65th, and 95th percentiles), which had the lowest AIC value, was selected. The restricted cubic spline, adjusted for age and sex, showed an S-shaped correlation between the Tyg index and MACE. The risk of MACE did not show a significant increase when the Tyg index was either below 8.715 or above 9.725. However, when the Tyg index was between 8.715 and 9.725, the risk of MACE significantly increased with an increase in the Tyg index (Fig. 3).

After univariate Cox regression, several factors emerged as predictive factors for MACE. These included age, weight, catheter ablation, hypertension, HF, stroke/TIA, systemic embolism, CAD, chronic obstructive pulmonary disease, OAC only, left atrial anterior-posterior diameter, LVD, right atrial maximum diameter, LVEF, glutamyl transpeptidase, serum uric acid, NT-pro BNP, high-density lipoprotein cholesterol, serum creatinine, and CHA2DS2-VASc score. Additionally, the Tyg index was associated with the risk of MACE, both as categorical data (HR: 2.05, 95%CI:1.65–2.56) and continuous data (HR: 2.01, 95%CI:1.65–2.45) (Additional file 1). After adjusting for CHA2DS2-VASc variables and catheter ablation, the Tyg index remained independently associated with MACE (as categorical data: HR: 1.91, 95% CI: 1.53–2.38; as continuous data: HR: 1.77, 95% CI: 1.44–2.17) (Additional file 2 and 3).

### Subgroup analysis

To explore the association between MACE and the Tyg index further, subgroup analysis was performed based on age, sex, AF type, catheter ablation, and HF (Additional file 4). Statistically significant correlations with MACE were detected in subgroups including males and females as well as individuals aged < 65 years and those aged ≥ 65 years. Regardless of whether the patient presented with paroxysmal or persistent AF, with or without HF, the Tyg index showed an association with MACE. In patients who did not undergo catheter ablation, the Tyg index was associated with the risk of MACE. However, in patients undergoing catheter ablation, the association between MACE and the Tyg index did not reach statistical significance (HR: 1.096, 95%CI: 0.507–2.365).

### Prediction performance of CHA2DS2-VASc plus Tyg Index Scoring System

**Fig. 4:**
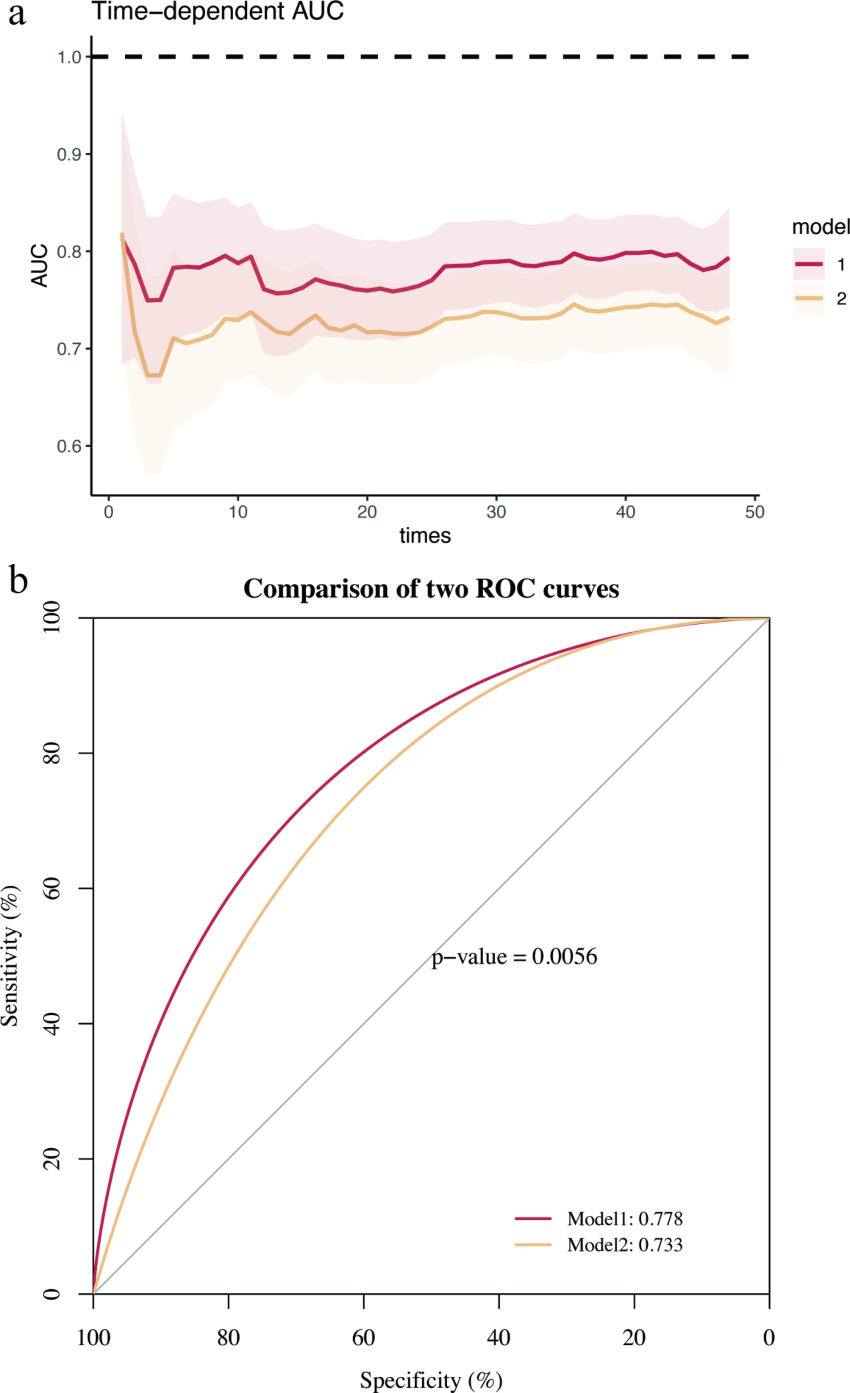
a: Time-dependent area under curve of CHA2DS2-VASc model with the addition of Tyg index versus traditional CHA2DS2-VASc model. b: Receiver Operating Characteristic curves compared the CHA2DS2-VASc model with the addition of Tyg index with traditional CHA2DS2-VASc model. Tyg index = triglyceride glucose index; AUC = area under curve; ROC = receiver operating characteristic; Model 1 = the CHA2DS2-VASc model with the addition of the Tyg index; Model 2 = the CHA2DS2-VASc model

**Fig. 5:**
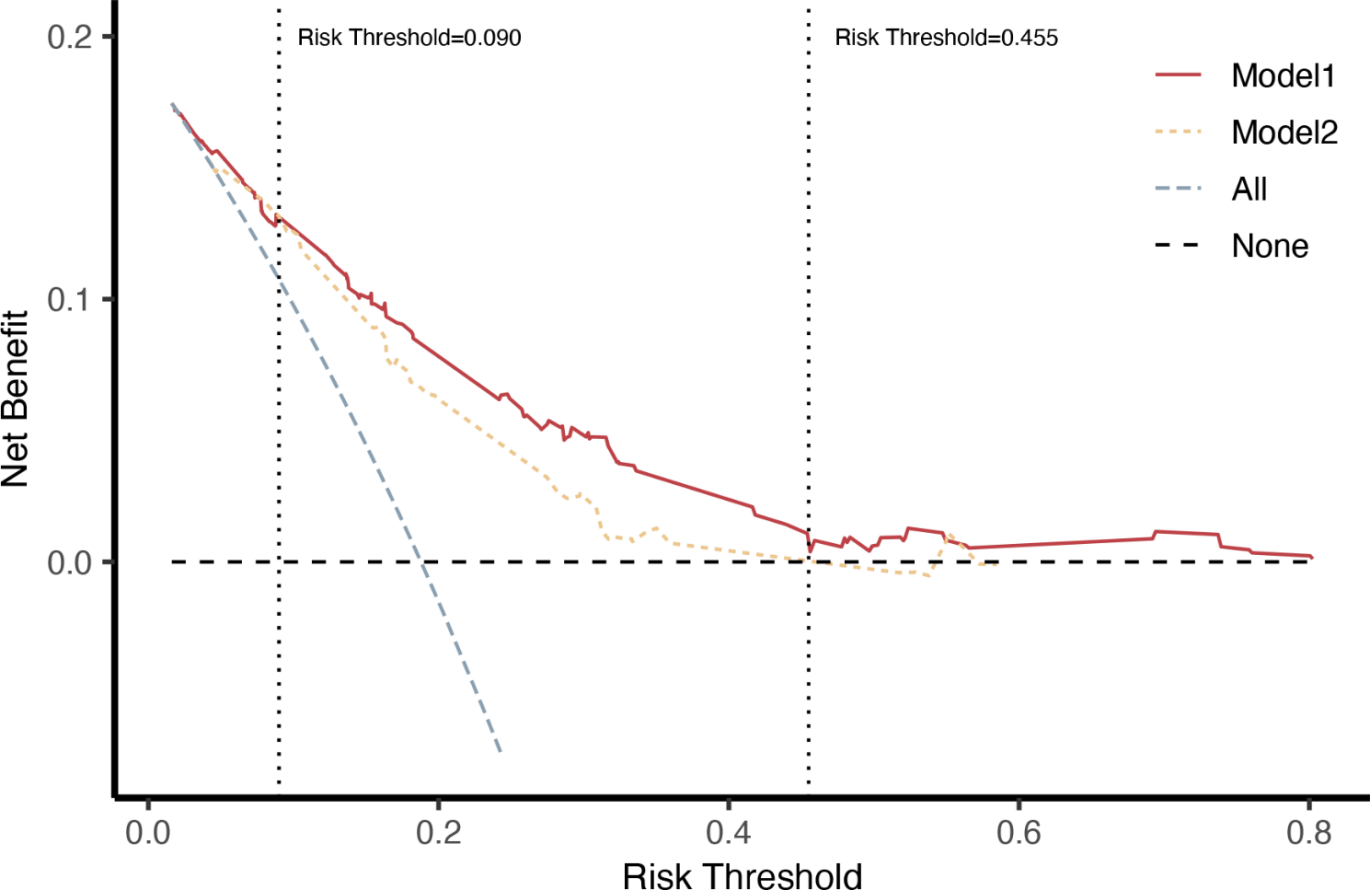
Decision curves of major adverse cardiovascular events prediction model. Model 1 = the CHA2DS2-VASc model with the addition of the Tyg index; Model 2 = the CHA2DS2-VASc model

The new MACE prediction model (model 1), which includes both the Tyg index and CHA2DS2-VASc variables, had a lower AIC in contrast to the traditional CHA2DS2-VASc model (model 2) (AIC for model 1: 1814.092, AIC for model 2:1855.675). The time-dependent AUC curve shows that the AUC of model 1 surpasses that of model 2 over the 48-month follow-up period (Fig. 4a). The two models showed significant differences as observed at the three time points: 12, 36, and 48 months (*P* = 0.030, 0.003, 0.040, respectively). However, at 24 months there was no significant difference (*P* = 0.111). Without considering the time variable, the ROC curves for both models were obtained. The AUC of model1 was 0.778, significantly higher than the AUC of model2, which stood at 0.733 (*P* = 0.0056) (Fig. 4b). The incorporation of the Tyg index into the CHA2DS2-VASc score resulted in a novel scoring system known as the CHA2DS2-VASc-TyG score. A score of ≥ 4 in this system indicates a higher risk of MACE (HR: 4.678, 95% CI: 3.278–6.675). The DCA indicates that model 1 provides a greater net benefit compared to both the intervention-for-all and intervention-for-none strategies and surpasses model 2 when the risk threshold is above 0.090. On the other hand, model 2’s net benefit was less than that of the intervention-for-none strategy at a risk threshold greater than 0.455 (Fig. 5).

## Discussion

This study revealed a significant correlation between MACE and the Tyg index in patients with AF without diabetes, independent of catheter ablation and CHA2DS2-VASc variables. Moreover, an S-shaped relationship was found between MACE and the Tyg index. Incorporating CHA2DS2-VASc variables and Tyg index into a new model improved fitting performance and enhanced predictive ability compared to the traditional CHA2DS2-VASc model, thus improving the risk assessment of MACE. The DCA shows that the new model offers superior assistance in clinical decision-making compared to the traditional model.

AF is currently the most common type of arrhythmia, exerting a significant impact on public health [1]. Insulin resistance, recognized as a crucial pathophysiological mechanism in the progression of obesity and diabetes, has considered to be associated with adverse cardiovascular prognosis [3]. Its association with AF occurrence and development is multifaceted, involving mechanisms that may be related to oxidative stress, epicardial adipose tissue, and cardiac fibrosis [4, 19, 20]. Epicardial adipose tissue and myocardial interstitial fibrosis disrupt myocardial electrical conduction and are related to myocardial electrical remodeling, thereby promoting AF generation and maintenance [21]. Additionally, these conditions increase the stiffness of the ventricular walls and reduce the contractile function of the ventricles [3]. Moreover, insulin resistance induces inflammation and facilitates the progression of inflammatory processes, which exacerbate endothelial cell damage and contribute to the formation and progression of atherosclerosis [22]. Furthermore, insulin resistance facilitates the inactivation of nitric oxide and reduces the sensitivity of platelets to antiaggregating actions [23]. Quantifying insulin resistance has traditionally posed a clinical challenge due to high cost and implementation difficulties. However, the Tyg index is one of the evaluation methods for insulin resistance, which is cheap but can achieve reliable detection efficiency [24]. The latest evidence has confirmed an association between the Tyg index and metabolic disorders including hyperlipidemia and hyperuricemia, underscoring their role as predictive risk factors for poor cardiovascular prognosis [25–27]. Moreover, a higher Tyg index increases the risk of ischemic stroke and myocardial infarction in patients, making it a potential indicator for predicting adverse cardiovascular outcomes [7, 9, 28]. Recent studies suggested an association between the occurrence of AF and the Tyg index, particularly among patients without diabetes [11]. Furthermore, patients with AF with a higher Tyg index level exhibit a higher recurrence rate following catheter ablation [10]. These findings collectively suggest that the Tyg index holds promise for improving risk assessment performance in patients with AF without diabetes.

In this cohort, participants with elevated Tyg levels had higher NT-pro BNP levels, lower LVEF, and a greater prevalence of comorbidities, including HF, stroke and CAD, all of which are correlated with adverse cardiovascular prognosis in patients with AF [1]. Furthermore, the high-Tyg group demonstrated elevated levels of serum creatinine and glutamyl transpeptidase, suggesting the Tyg index’s potential as a feasible biomarker for various metabolic diseases, such as hyperlipidemia, hyperuricemia, and chronic kidney disease. Thus, it serves as an indicator of the complex metabolic and inflammatory status of the participants [29, 30].

This study revealed that the high-Tyg group had a higher risk of MACE compared to the groups with low- and medium- Tyg index. The Tyg index remained independently associated with MACE risk even after adjusting for traditional CHA2DS2-VASc variables and catheter ablation. Moreover, while no significant difference in MACE risk was found between the low-Tyg and medium-Tyg groups over the 48-month follow-up, a landmark analysis of the last 24 months indicated a higher risk for those in the medium-Tyg group than those in the low-Tyg group, underscoring the Tyg index’s long-term predictive value.

Hu et al. [31] reported an association between lower Tyg-body mass index (Tyg-BMI) and increased all-cause mortality risk in AF patients over a 12-month follow-up. The formula for calculating Tyg-BMI mainly depends on the body mass index rather than the Tyg index. Given that their cohort were all severely ill patients with numerous comorbidities, patients with a higher body mass index likely exhibited stronger tolerance for disease and medication, impacting their findings. A study reported by Liu et al. [32] showed that AF with lower body mass is significantly associated with poor cardiovascular prognosis, which also supports this viewpoint. And the research outcome of Hu et al. focused on all-cause mortality, some causes of death may not significantly correlate with Tyg status.

Nonetheless, appropriate lifestyle improvements can mitigate the incidence of adverse events in patients with AF [33]. The Tyg index offers an objective reference for determining the timing and necessity of lifestyle adjustments. The restricted cubic spline plot suggests that patients with a Tyg index between 8.715 and 9.725 may benefit from dietary control and lifestyle adjustments to significantly reduce cardiovascular and cerebrovascular risks. Patients with a Tyg index > 9.725 require more strict lifestyle management to improve their metabolic status and lower their Tyg index and may require more follow-up visits and health guidance to lower their cardiovascular and cerebrovascular risks.

Catheter ablation is currently considered a first-line treatment for AF, which not only reduces palpitation occurrence in patients but also improves their long-term prognosis [1]. In the catheter ablation subgroup, no significant association was found between MACE and the Tyg index. Due to the patient selection for catheter ablation, most of these patients were younger (mean age: 59.7 years) and had fewer comorbidities and a lower Tyg index; only 11 (3.6%) patients experienced MACE during the 4-year follow-up, which all influenced the results. In addition, the subgroup analysis revealed a stronger correlation between MACE risk and the Tyg index among patients aged under 65 years old, suggesting potential directions for future research. Sex hormones have long been associated with glucose and lipid metabolism, cardiovascular disease risk, and cardiovascular prognosis [34, 35]. Nevertheless, the subgroup analysis did not show significant sex-based differences in the predictive performance of the Tyg index for MACE. This observation could be due to the higher average age of participating women (69.5 years old), many of whom have already entered menopause, thereby reducing the impact of hormones on outcomes. Existing literature has confirmed the Tyg index as a reliable predictor of cardiovascular prognosis across different races [36, 37]. However, further studies are needed to validate its predictive performance, specifically in patients with AF from different races.

In contemporary clinical practice, cardiologists frequently rely on the CHA2DS2-VASc scoring system to predict cardiovascular and cerebrovascular risks in patients with AF [1, 38]. However, this scoring system, comprising demographic characteristics and clinical conditions, has been criticized for its limited predictive performance and calls for updates [39, 40]. Despite numerous attempts to develop new models, many prove either too complex to use or have inadequate predictive performance [41]. Therefore, a novel CHA2DS2-VASc prediction model incorporating the Tyg index based on Cox regression was established. This model accounts for patients’ comprehensive metabolic status and demonstrates superior MACE prediction performance in patients with AF without diabetes compared to the traditional model. The novel CHA2DS2-VASc-Tyg scoring system demonstrates that patients with a score ≥ 4 have a poorer cardiovascular prognosis than those with a score < 4. Furthermore, the DCA confirms the practicality of the new model with auxiliary clinical decision-making ability [18]. Given the ready availability of the Tyg index, the new model can achieve good predictive ability without the need for excessive examination. Patients assessed as high-risk by the model during hospitalization can be recommended an increase in the frequency of follow-ups. During the follow-ups, their cardiovascular event risk can be re-stratified based on changes in the Tyg index, enabling physicians to promptly obtain their current prognostic status. This allows for more composed and objective guidance in clinical decision-making.

### Strengths and limitations

This study analyzes the predictive capacity of the Tyg index for cardiovascular prognosis in AF patients without diabetes. Furthermore, it establishes a novel prediction system that outperforms the widely recognized prognosis prediction model, CHA2DS2-VASc. This novel system is characterized by its convenience, reliability, and suitability for clinical application. However, this study has some limitations. First, it was a single-center retrospective cohort study involving 864 medium-sized participants. Although relevant statistical methods, including subgroup analysis, were used to control potential confounding factors, this may not have been sufficient to reflect the causal relationship between Tyg and MACE. Second, the Tyg index is easily influenced by other factors, and patients’ metabolic status may be better assessed by monitoring Tyg fluctuations during follow-up. Last, this study population was entirely Chinese, lacking data on populations of other races.

## Conclusions

The high Tyg index correlates with poor cardiovascular prognosis in patients with AF without diabetes. A new MACE prediction model, incorporating the Tyg index and CHA2DS2-VASc variables, has enhanced prediction efficiency, and holds promise for guiding clinical decision-making. This model is expected to become a reliable tool for estimating the cardiovascular risk of patients with AF without diabetes.

## Electronic supplementary material

Below is the link to the electronic supplementary material.

Supplementary Material 1

Supplementary Material 2

Supplementary Material 3

Supplementary Material 4

Supplementary Material 5

Supplementary Material 6

## Data Availability

The datasets used and/or analyzed during the current study are available from the corresponding author on resonable request.

## Declarations

### Supplementary Information

Supplementary Material 1: Additional file 1: Univariate cox regression for all variables. HR: hazard ratio; AF: atrial fibrillation; CHA2DS2-VASc: Heart failure, hypertension, diabetes, coronary artery disease/peripheral artery disease and female with one point each, one point for 65–74 years old, and two points for ≥ 75 years old, stroke/transient ischemic attack/systemic embolism with 2 points; TIA: transient ischemic attack; COPD: chronic obstructive pulmonary disease; GGT: glutamyl transpeptidase; LDL: low density lipoprotein; HDL: high density lipoprotein; SUA: serum uric acid; SCR: serum creatinine; NT-pro BNP: N-terminal pro B-type natriuretic peptide; LAD: left atrial anterior-posterior diameter; LVD: left ventricular maximum diameter; RAD: right atrial maximum diameter; RVD: right ventricular maximum diameter; LVEF: left ventricular ejection fraction; OAC: oral anticoagulation; ARNI: Angiotensin Receptor & Neprilysin Inhibitor; ACEI: angiotensin converting enzyme inhibitor; ARB: angiotensin receptor blocker; Tyg: triglyceride glucose index.

Supplementary Material 2: Additional file 2: Multivariate cox regression model for Tyg index (as categorical data) adjusting for CHA2DS2-VASc variables and catheter ablation. TIA: transient ischemic attack; CAD: coronary artery disease; PAD: peripheral artery disease; Tyg: triglyceride glucose index; HR: hazard ratios.

Supplementary Material 3: Additional file 3: Multivariate cox regression model for Tyg index (as continuous data) adjusting for CHA2DS2-VASc variables and catheter ablation. TIA: transient ischemic attack; CAD: coronary artery disease; PAD: peripheral artery disease; Tyg: triglyceride glucose index; HR: hazard ratio.

Supplementary Material 4: Additional file 4: Forest plot for subgroup analysis. AF = atrial fibrillation.

Supplementary Material 5: Additional file 5: Editing certificate.

Supplementary Material 6: Additional file 6: Similarity check report.

## Abbreviations

AF: Atrial Fibrillation
AIC: Akaike Information Criterion
AUC: Area Under Curve
CAD: Coronary Artery Disease
CHA2DS2-VASc: Congestive Heart Failure, Hypertension, Age ≥ 75 years, Diabetes Mellitus, Stroke/Transient Ischemic Attack/Thromboembolism, Vascular Disease, Age 65-74 years, Sex Category (Female)
CI: Confidence Interval
DCA: Decision Curve Analysis
HF: Heart Failure
HOMA: IR: Homeostatic Model Assessment of Insulin Resistance
HR: Hazard Ratio
LVD: Left Ventricular Dysfunction
LVEF: Left Ventricular Ejection Fraction
MACE: Major Adverse Cardiovascular Events
NT: pro BNP-N-terminal pro B-type natriuretic peptide
OAC: Oral Anticoagulant
PAD: Peripheral Artery Disease
ROC: Receiver Operating Characteristic
TIA: Transient ischemic attack
Tyg: Triglyceride-Glucose
Tyg: BMI-Triglyceride-Glucose Body Mass Index
