# Supplementary material for: Association between triglyceride glucose index and adverse cardiovascular prognosis in patients with atrial fibrillation without diabetes: a retrospective cohort study
Source: Lipids Health Dis. 2025 Jan 25;24:23. doi: 10.1186/s12944-025-02447-3 (PMC11762522; doi:10.1186/s12944-025-02447-3)
Supplement: Supplementary file 1 — Supplementary Material 1 [file 12944_2025_2447_MOESM1_ESM.docx]

**Additional file 1: Univariate cox regression for all variables.**

|  | **HR** | **95% Confidence interval** | ***P*.value** |
| --- | --- | --- | --- |
|  |  |  |  |
| Age ≥ 65 years old | 4.577 | 2.909-7.202 | <0.001 |
| Female sex | 1.040 | 0.753-1.437 | 0.813 |
| Persistent AF | 1.260 | 0.913-1.740 | 0.160 |
| Catheter ablation | 0.119 | 0.064-0.220 | <0.001 |
| Weight, kg | 0.982 | 0.969-0.996 | 0.012 |
| Height, m | 0.807 | 0.117-5.585 | 0.828 |
| Systolic pressure, mmHg | 1.001 | 0.993-1.008 | 0.883 |
| Diastolic pressure, mmHg | 0.991 | 0.979-1.003 | 0.148 |
| CHA2DS2-VASc score | 1.449 | 1.333-1.575 | <0.001 |
| Comorbidities |  |  |  |
| Hypertension | 1.439 | 1.039-1.992 | 0.028 |
| Heart failure | 2.517 | 1.822-3.478 | <0.001 |
| Stroke/TIA | 2.087 | 1.460-2.985 | <0.001 |
| Systemic embolism | 3.035 | 1.488-6.190 | 0.002 |
| Coronary artery disease | 1.677 | 1.190-2.361 | 0.003 |
| Peripheral artery disease | 0.973 | 0.496-1.909 | 0.936 |
| COPD | 1.618 | 1.009-2.593 | 0.046 |
| Sleep disorders | 0.917 | 0.376-2.238 | 0.850 |
| Hyperthyroidism | 0.678 | 0.168-2.738 | 0.585 |
| Hypothyroidism | 0.872 | 0.357-2.127 | 0.763 |
| Laboratory tests: |  |  |  |
| GGT, IU/L | 1.002 | 1.001-1.003 | <0.001 |
| Cholesterol, mmol/L | 0.917 | 0.777-1.082 | 0.303 |
| LDL, mmol/L | 0.925 | 0.759-1.128 | 0.442 |
| HDL ≥ 1 mmol/L | 0.410 | 0.297-0.566 | <0.001 |
| SUA, umol/L | 1.002 | 1.001-1.004 | <0.001 |
| SCR, umol/L | 1.002 | 1.001-1.003 | <0.001 |
| NT-pro BNP, ng/L | 1.000 | 1.000-1.000 | <0.001 |
| Echocardiogram data: |  |  |  |
| LAD, mm | 1.040 | 1.024-1.057 | <0.001 |
| LVD, mm | 1.047 | 1.026-1.069 | <0.001 |
| RAD, mm | 1.045 | 1.025-1.065 | <0.001 |
| RVD, mm | 0.998 | 0.940-1.058 | 0.938 |
| LVEF, % | 0.973 | 0.961-0.985 | <0.001 |
| Medications |  |  |  |
| OAC only | 0.545 | 0.395-0.752 | <0.001 |
| Antiplatelet only | 1.628 | 0.994-2.666 | 0.053 |
| OAC+ Antiplatelet | 0.843 | 0.443-1.601 | 0.601 |
| ARNI/ACEI/ARB: | 0.997 | 0.674-1.475 | 0.989 |
| β-blocker: | 1.263 | 0.902-1.767 | 0.174 |
| Statin: | 1.009 | 0.706-1.442 | 0.962 |
| Tyg(continuous) | 2.012 | 1.652-2.452 | <0.001 |
| Tyg(categorical) | 2.052 | 1.646-2.559 | <0.001 |

HR: hazard ratio; AF: atrial fibrillation; CHA2DS2-VASc: Heart failure, hypertension, diabetes, coronary artery disease/peripheral artery disease and female with one point each, one point for 65-74 years old, and two points for ≥ 75 years old, stroke/transient ischemic attack/systemic embolism with 2 points; TIA: transient ischemic attack; COPD: chronic obstructive pulmonary disease; GGT: glutamyl transpeptidase; LDL: low density lipoprotein; HDL: high density lipoprotein; SUA: serum uric acid; SCR: serum creatinine; NT-pro BNP: N-terminal pro B-type natriuretic peptide; LAD: left atrial anterior-posterior diameter; LVD: left ventricular maximum diameter; RAD: right atrial maximum diameter; RVD: right ventricular maximum diameter; LVEF: left ventricular ejection fraction; OAC : oral anticoagulation; ARNI: Angiotensin Receptor & Neprilysin Inhibitor; ACEI: angiotensin converting enzyme inhibitor; ARB: angiotensin receptor blocker; Tyg: triglyceride glucose index
