# Supplementary material for: Association between triglyceride glucose index and adverse cardiovascular prognosis in patients with atrial fibrillation without diabetes: a retrospective cohort study
Source: Lipids Health Dis. 2025 Jan 25;24:23. doi: 10.1186/s12944-025-02447-3 (PMC11762522; doi:10.1186/s12944-025-02447-3)
Supplement: Supplementary file 2 — Supplementary Material 2 [file 12944_2025_2447_MOESM2_ESM.docx]

**Additional file 2: Multivariate cox regression model for Tyg index (as categorical data) adjusting for CHA2DS2-VASc variables and catheter ablation.**

|  | **HR** | **95% Confidence interval** | **p.value** |
| --- | --- | --- | --- |
|  |  |  |  |
| Age ≥ 65 years old | 3.20 | 1.98-5.15 | <0.001 |
| Female sex | 0.81 | 0.58-1.13 | 0.210 |
| Hypertension | 1.02 | 0.73-1.44 | 0.890 |
| Heart failure | 1.71 | 1.22-2.40 | 0.002 |
| Stroke/TIA/Systematic embolism | 1.58 | 1.10-2.26 | 0.014 |
| CAD/PAD | 0.79 | 0.56-1.12 | 0.180 |
| Catheter ablation | 0.25 | 0.13-0.48 | <0.001 |
| Tyg (categorical) | 1.91 | 1.53-2.38 | <0.001 |

TIA: transient ischemic attack; CAD: coronary artery disease; PAD: peripheral artery disease; Tyg: triglyceride glucose index; HR: hazard ratios.
