# Supplementary material for: Association between triglyceride glucose index and adverse cardiovascular prognosis in patients with atrial fibrillation without diabetes: a retrospective cohort study
Source: Lipids Health Dis. 2025 Jan 25;24:23. doi: 10.1186/s12944-025-02447-3 (PMC11762522; doi:10.1186/s12944-025-02447-3)
Supplement: Supplementary file 3 — Supplementary Material 3 [file 12944_2025_2447_MOESM3_ESM.docx]

**Additional file 3: Multivariate cox regression model for Tyg index (as continuous data) adjusting for CHA2DS2-VASc variables and catheter ablation.**

|  | **HR** | **95% Confidence interval** | **p.value** |
| --- | --- | --- | --- |
|  |  |  |  |
| Age ≥ 65 years old | 3.22 | 1.99-5.18 | <0.001 |
| Female sex | 0.84 | 0.60-1.18 | 0.320 |
| Hypertension | 1.09 | 0.78-1.54 | 0.610 |
| Heart failure | 1.64 | 1.17-2.31 | 0.004 |
| Stroke/TIA/Systematic embolism | 1.47 | 1.03-2.11 | 0.037 |
| CAD/PAD | 0.85 | 0.60-1.21 | 0.360 |
| Catheter ablation | 0.25 | 0.13-0.49 | <0.001 |
| Tyg (continuous) | 1.77 | 1.44-2.17 | <0.001 |

TIA: transient ischemic attack; CAD: coronary artery disease; PAD: peripheral artery disease; Tyg: triglyceride glucose index; HR: hazard ratios
