# Supplementary figures and images for: Association between triglyceride glucose index and adverse cardiovascular prognosis in patients with atrial fibrillation without diabetes: a retrospective cohort study
Source: Lipids Health Dis. 2025 Jan 25;24:23. doi: 10.1186/s12944-025-02447-3 (PMC11762522; doi:10.1186/s12944-025-02447-3)

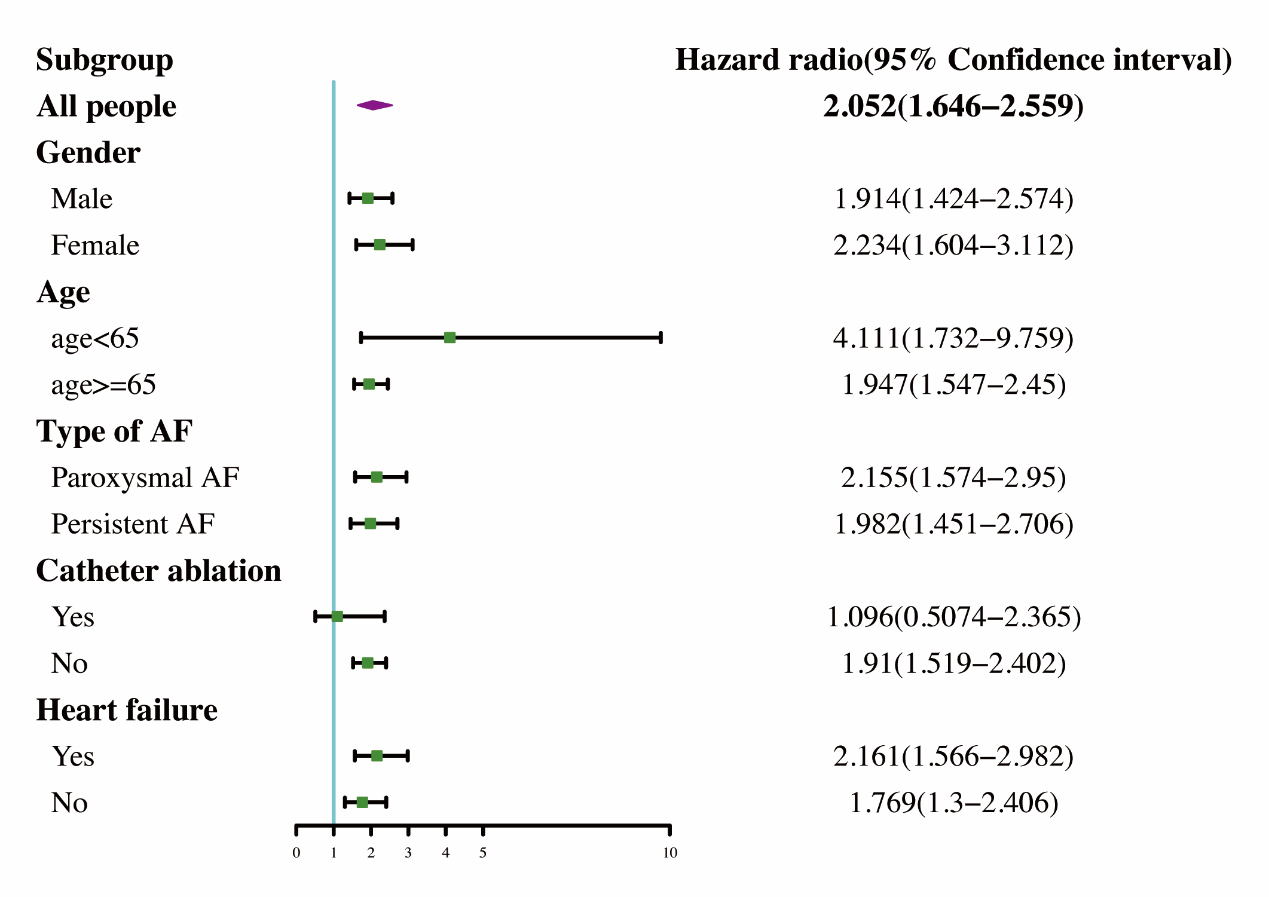
Additional file4: Forest plot for subgroup analysis.

AF= atrial fibrillation

Supplement: Supplementary file 4 — Supplementary Material 4 [file 12944_2025_2447_MOESM4_ESM.docx]
